# A Hybrid Machine-Learning-Based Method for Analytic Representation of the Vocal Fold Edges during Connected Speech

**DOI:** 10.3390/app11031179

**Published:** 2021-01-27

**Authors:** Ahmed M. Yousef, Dimitar D. Deliyski, Stephanie R. C. Zacharias, Alessandro de Alarcon, Robert F. Orlikoff, Maryam Naghibolhosseini

**Affiliations:** 1 Department of Communicative Sciences and Disorders, Michigan State University, East Lansing, MI 48824, USA; 2 Department of Communicative Sciences and Disorders, Michigan State University, East Lansing, MI 48824, USA; 3 Head and Neck Regenerative Medicine Program, Mayo Clinic, Scottsdale, AZ 85259, and Department of Otolaryngology-Head and Neck Surgery, Mayo Clinic, Phoenix, AZ 85054, USA; 4 Division of Pediatric Otolaryngology, Cincinnati Children’s Hospital Medical Center, Cincinnati, OH 45229, and Department of Otolaryngology—Head and Neck Surgery, University of Cincinnati College of Medicine, Cincinnati, OH 45267, USA; 5 College of Allied Health Sciences, East Carolina University, Greenville, NC 27834, USA; 6 Department of Communicative Sciences and Disorders, Michigan State University, East Lansing, MI 48824, USA

**Keywords:** high-speed videoendoscopy, connected speech, automated machine-learning-based edge detection

## Abstract

Investigating the phonatory processes in connected speech from high-speed videoendoscopy (HSV) demands the accurate detection of the vocal fold edges during vibration. The present paper proposes a new spatio-temporal technique to automatically segment vocal fold edges in HSV data during running speech. The HSV data were recorded from a vocally normal adult during a reading of the “Rainbow Passage.” The introduced technique was based on an unsupervised machine-learning (ML) approach combined with an active contour modeling (ACM) technique (also known as a hybrid approach). The hybrid method was implemented to capture the edges of vocal folds on different HSV kymograms, extracted at various cross-sections of vocal folds during vibration. The k-means clustering method, an ML approach, was first applied to cluster the kymograms to identify the clustered glottal area and consequently provided an initialized contour for the ACM. The ACM algorithm was then used to precisely detect the glottal edges of the vibrating vocal folds. The developed algorithm was able to accurately track the vocal fold edges across frames with low computational cost and high robustness against image noise. This algorithm offers a fully automated tool for analyzing the vibratory features of vocal folds in connected speech.

## 1. Introduction

The endoscopic imaging of the laryngeal anatomy and phonatory function has played a central role in the instrumental clinical voice assessment [1–4]. At present, the most common clinical technique employed in laryngeal imaging is videostroboscopy [5–7]. The coupling between the stroboscopic unit and the video camera in videostroboscopy offers slow-motion visualization of the laryngeal structures and vocal fold vibrations during phonation. However, videostroboscopy is only capable of evaluating vibratory behavior during sustained vocalizations, i.e., uttering prolonged vowels [8–11]. Additionally, due to the low sampling rate of the camera, videostroboscopy is incapable of capturing the cycle-to-cycle and intra-cycle details of vocal fold vibration, which is critical when those vibrations are aperiodic, a common occurrence in vocal disorders [12,13]. High-speed videoendoscopy (HSV) is an advanced laryngeal imaging technique that overcomes the latter limitation by recording the intra-cycle vibratory characteristics of the vocal folds at high frame rates without depending on the periodicity of the acoustic voice signal [12–14]. Moreover, through a recent advancement, HSV can be used to objectively measure vocal fold vibratory characteristics with high temporal resolution not only in sustained phonation but also in running speech [15–20]. Hence, HSV is an effective tool that can improve our understanding of complex physiological and phonatory mechanisms of voice production in ways that are not feasible when using videostroboscopy [12,13,21,22].

Since voice disorders are often revealed in connected speech [23–28], using HSV in voice assessment can serve as a powerful tool in studying the cycle-to-cycle and intra-cycle vibratory details besides the non-stationary events (e.g., voice breaks, and voicing onsets and offsets) during phonation in connected speech [15,19,29–31]. However, in clinical settings, it is not feasible to navigate through the huge amount of data obtained using HSV without the aid of automated analysis techniques. The development of automated methods for extracting HSV-based measures of vocal fold vibration enables us to acquire clinically relevant vocal fold vibratory characteristics during running speech [32,33]. The mining of massive HSV datasets requires specialized machine-learning (ML) methods. Using ML, we can classify similar and dissimilar structures and/or discover hidden patterns in the data efficiently with a low computational cost.

To study the vibratory characteristics of the vocal folds in connected speech, it is essential to develop algorithms for the detection of the vibrating vocal fold edges during phonation. Developing fully automated spatial segmentation algorithms for such an edge-detection task will facilitate extraction of HSV-based measures during connected speech. Various spatial segmentation methods have been implemented to detect the glottal edges in HSV data in isolated sustained vowels [36–40], e.g., histogram thresholding [38,41,42], seeded region growing procedures [37,43,44], level set methods [45,46], watershed transform [47], and active contour models [39,40,48,49]. Four recent ML techniques based on deep learning have successfully segmented the glottis/vocal fold edges in HSV recordings with high accuracy [32–35]. The HSV datasets in these studies were recorded during the production of sustained phonation, and not running speech. Since these approaches utilized a supervised learning framework (i.e., deep neural networks), they required manual annotation of the vocal fold edges in HSV frames in order to be used as a training dataset.

Recently, we introduced a fully automated method to segment the glottal area using HSV during connected speech for the first time [20]. This method applied a spatio-temporal approach based on active-contour modeling (ACM), which enabled us to detect the glottal edges during vocal fold vibration. ACM is an iterative energy minimization technique for edge detection, and it requires initializing a deformable contour near the edges of interest in an image [50]. One of the benefits of ACM is that it is not noise sensitive [50–52], unlike some of the other spatial segmentation methods, which are more vulnerable to noise and intensity homogeneity in the image [39,43,45,53,54]. In our proposed algorithm, the segmentation was applied to HSV kymograms at different vocal fold cross-sections in individual vocalizations to capture the glottal edges in each kymogram. The detected edges in the kymograms were registered back to the HSV frames [20]. The method we developed was able to detect the glottal edges in 88% of the vocalizations. The algorithm was not successful when the kymograms had very dim lighting. Although ACM is less vulnerable to noise in the image, it is sensitive to the initialization of the contour. The initial contour should be close to the glottal edge in order to have the best performance of ACM, which is a limitation of this method. Moreover, since ACM is an iterative method, it required a relatively long time for convergence because the analysis is done at all cross-sections of the vocal folds for each vocalization. ML may overcome the limitation of ACM on its dependency to the contour initialization and the high computational cost.

In this work, we propose a hybrid method based on an unsupervised ML (clustering) technique and ACM for vocal fold edge representation in HSV data during connected speech. This hybrid approach can provide a more robust spatial segmentation performance with less computational costs than the ACM method alone. Data clustering is a way of grouping data with similar characteristics, which is used extensively in image analysis [55]. Clustering is based on partitioning the data into clusters of data points that have similar features within each cluster and dissimilarity with other clusters [56]. In this project, the clustering was used to group the pixels belonging to the glottal area into one cluster and the rest of the pixels into another cluster. Clustering is considered an unsupervised ML method since the data points are not required to be labeled, meaning we do not need to visually label the pixels to indicate whether they belong to the glottal area or not, a time-consuming process involving manual analysis of the data. Therefore, our developed method in this work is fully automated and does not require the user intervention in the data analysis. The clustering technique can perform efficiently in detecting the glottal area in HSV data. This is because the glottal area is relatively dark and can be silhouetted against the brighter surrounding tissues of the vocal folds [32]. In the present study, the k-means clustering method is combined with ACM as a shape-based image segmentation method to improve our previously introduced method (i.e., ACM) [20]. As such, the clustering technique is utilized to obtain an accurate initialization contour close to the glottal area, which was given as an input to the ACM method. The ACM algorithm is used to capture the accurate glottal edges during the vocal fold vibrations. The goals of this study are: (i) to present the developed theoretical framework for the proposed method, (ii) to exhibit its feasibility in vocal fold edge representation in HSV data during connected speech, and (iii) to show its robustness for challenging color HSV images. Hence, the developed method was applied to the HSV data obtained from a vocally normal adult using a color high-speed camera.

## 2. Materials and Methods

### 2.1. Clinical Data

A custom-built color HSV system was used to record a 38-year-old female during recitation of the “Rainbow Passage.” The participant was vocally normal without any history of voice disorders. The examination was conducted at the Center for Pediatric Voice Disorders, Cincinnati Children’s Hospital Medical Center and approved by the Institutional Review Board. A FASTCAM SA-Z color high-speed camera (Photron Inc., San Diego, CA, USA) with a 12-bit color image sensor and 64 GB of cache memory, set at 4000 frames per second and 249 μs integration time, was used to obtain the data. The camera was coupled with a 3.6 mm Olympus ENF-GP Fiber Rhinolaryngoscope (Olympus Corporation, Tokyo, Japan) and a 300 W xenon light source, model 7152A (PENTAX Medical Company, Montvale, NJ, USA). The recording length was 29.14 s (116,543 video frames) with image frame resolution of 256 × 256 pixels. The recorded video was saved as an uncompressed 24-bit RGB AVI file.

### 2.2. Data Analysis

Several pre-processing steps were applied to the HSV data before proceeding with the proposed spatial segmentation approach. Temporal segmentation [15] and motion compensation [18] were first applied sequentially to automatically extract the vocalized segments of the HSV recordings and capture the location of the moving vocal folds across the frames. HSV kymograms were extracted next at different intersections of the vocal folds. After applying the pre-processing steps, the clustering technique was implemented on each kymogram to initialize a contour line based on the captured glottal area. The ACM method was applied to the kymograms to complete the segmentation task. The segmented glottal edges in the kymograms were registered back to the HSV frames to detect the glottal edges in each individual HSV frame. The algorithms were implemented using the 64-bit MATLAB R2019a (MathWorks Inc., Natick, MA, USA).

#### 2.2.1. Data Preprocessing

The temporal segmentation, a technique previously developed in our lab, was used to automatically extract the timestamps of the vibratory onsets and offsets of the vocalized segments in the HSV recording [15]. Subsequently, a denoising algorithm and motion compensation were implemented across the video frames of each vocalization to track the location of the vocal folds inside a window—encompassing the vibrating vocal folds [18,57]. The HSV frames were then cropped based on the center and size of the motion window. The kymograms were extracted next at various cross sections along the anteroposterior length of the vocal folds from the cropped HSV frames for each vocalization. The y-axis of the kymogram image represents the left-right dimension of the video frame, while the x-axis refers to time (frame number).

Each kymogram was smoothed using a moving average 1D filter with a window size of 5 pixels (along the y-axis) to mitigate the impact of noise in the images. Therefore, each pixel intensity in the kymogram was calculated by taking the average intensity of the four neighborhood pixels (two pixels above and below the pixel of interest). Since our area of interest, i.e., the glottal area, was located in the middle section of the kymograms, a Tukey window function (window size of 15 pixels) was applied next to provide higher weights to the pixels located in the middle and less weights to the pixels located in the top and bottom of the kymograms. With these preprocessing steps, the kymograms became ready for the feature extraction, explained below.

#### 2.2.2. Feature Selection and Extraction

Selection of the right features is an essential step toward the successful implementation of the ML method. Extracting the features from an image mostly depends on the intensities and the texture of the pixels. The number of pixels in the horizontal and vertical directions in the kymogram comprises a 2-D matrix. Each cell in the matrix (pixel) comprises three image components with a numerical value ranging between 0 and 255, corresponding to the three color channels (i.e., red, green, and blue). The features were calculated based on the intensity values of only the red and green channels. The blue channel was excluded from the analysis due to the high noise level and the absence of essential information. In this work, three features were extracted, namely, two intensity features and a gradient feature. Different number and combinations of the aforementioned features were utilized in the development of the proposed algorithms to determine which features should be used to perform an accurate vocal fold edge representation.

##### Intensity Features:

The pixel intensities of the red and green channels in a kymogram were considered as two features. Since the regions of interest in the kymogram (glottal areas) have lower intensities (darker) than the neighborhood regions, selecting the pixel intensities as a feature was essential to facilitate distinguishing the glottal area from the laryngeal tissues in the kymograms. However, relying only on the intensities as features was not enough to segment the image because of the high level of noise in the present video data and appearance of dark pixels in places other than the glottis.

##### Gradient Feature:

The image gradient can be used to detect the glottal area edges given the contrast between the intensity of the glottis and the surrounding regions. Hence, the kymogram image gradient was used for feature extraction. The positive and negative gradients were computed along the x- and y-axis in the kymogram with a step size of 8 pixels. In the negative gradient, the pixels with positive values were assigned a value of zero and vice versa. An overall gradient magnitude was calculated by taking the square root of the sum of squared of the four negative and positive gradients in the two directions.

#### 2.2.3. Unsupervised Clustering Method

An unsupervised ML technique was implemented using the well-known k-means clustering algorithm for image segmentation [58]. The k-means clustering technique is based on partitioning a dataset into a certain number of disjoint clusters (groups of data). This technique requires the initialization of the number of clusters (k) and the center of each cluster (centroid). In this study, the number of clusters was selected to be two (inside or outside the glottal area) and the initial centroids were chosen based on the k-means++ algorithm, which uses a heuristic in order to initialize centroid seeds for k-means clustering (see [59] for the full details of the algorithm). The clustering algorithm then computed the distance between the centroids and each pixel in the kymogram. The distance was calculated using the Euclidean distance as follows:

(1) $$\text{d}=\left\|\text{I}\left(\text{x},\text{y}\right)-{\text{c}}_{\text{k}}\right\|,$$

where d is the Euclidean distance, I(x, y) corresponds to the intensity of the kymogram, x and y refer to the pixel coordinates, c_k_ is the cluster centroid, and k is the cluster number. Each pixel in the image was assigned to the nearest centroid based on the calculated distance leading to the formation of the initial clusters. Once the grouping was done, the algorithm recomputed the updated centroid of each cluster (c_k_) as follows:

(2) $${\text{c}}_{\text{k}}=\frac{1}{\text{k}}\sum_{\text{x}\in {\text{c}}_{\text{k}}}\sum_{\text{y}\in {\text{c}}_{\text{k}}}\text{I}\left(\text{x},\text{y}\right),$$

where this new centroid was the data point to which the summation of the distances from all the pixels located in that cluster was minimal. This process was repeated iteratively—reshaping the clusters in the image at each iteration—until converging, when the distance between the new and original centroids did not change.

Since the k-means clustering technique used the Euclidean distance measure, normalizing the features was necessary. Hence, the three extracted features were normalized between 0 and 1 before applying the clustering method. Instead of applying the clustering algorithm to the entire kymogram for a vocalization, each kymogram was divided into smaller kymograms with a maximum of 50 frames to mitigate any possible impact of the image noise on the clustering accuracy. For example, when part of the kymogram had extremely bright pixels (saturated or near-saturated pixels), the clustering technique may be misguided, particularly with a large number of frames. This might occur due to the movements of the epiglottis and large reflections from its surface.

After applying the clustering algorithm to each kymogram, each pixel in the kymogram was assigned to either cluster one or two. All the pixels in the same cluster had similar labels. Accordingly, a new binary labeled image of the kymogram was constructed, where each pixel had the binary value of the cluster number. To identify the label associated with the glottal area cluster in the labeled image, a procedure was developed based on computing the first moment of inertia of the original kymogram image. The first moment of inertia, denoted by M_1_ (y, n_i_) for each kymogram n_i_, was calculated as follows [20, 60]:

(3) $${\text{M}}_{1}\left(\text{y},{\text{n}}_{\text{i}}\right)=\frac{\sum_{\text{x}=1}^{{\text{K}}_{\text{w}}}\sum_{\text{y}=1}^{{\text{K}}_{\text{h}}}\text{I}\left(\text{x},\text{y},{\text{n}}_{\text{i}}\right)\text{y}}{\sum_{\text{x}=1}^{{\text{K}}_{\text{w}}}\sum_{\text{y}=1}^{{\text{K}}_{\text{h}}}\text{I}\left(\text{x},\text{y},{\text{n}}_{\text{i}}\right)},$$

where K_w_ is the image width (number of frames), and K_h_ is the kymogram image height. The first moment of inertia was computed for the green channel, which was less noisy compared to the other channels due to the Bayer filter decomposition. The moment of inertia was applied on an inverted kymogram to approximate a horizontal line passing through the center of the glottis (center of darkness). After obtaining the moment line of the original kymogram, the label associated with the glottal area cluster was determined from the binary labeled image of the kymogram. As such, the mode of the two binary values (1 and 2) in the labeled kymogram was computed for pixels located within 12 pixels above and below the moment line returning either one or two. If, for example, the mode returns a label value of one, then the label of the glottis cluster would be two. The algorithm then searched for the label of the glottis cluster between 7 pixels above and below the moment line in the binary labeled kymogram until the algorithm found all the pixels that belonged to the glottal area. The spatial location of the glottal edges corresponding to the left and right vocal fold were determined. Subsequently, the splines were used as the initial contours for the ACM method.

#### 2.2.4. Spatial Segmentation: The Hybrid Method

In this study, a hybrid method was developed by combining the unsupervised clustering technique (see Section 2.2.3) with an ACM method (see [20] for the complete description and details of implementing the ACM). The active contour in the ACM method is a spline that deforms spatially based on an internal rule (depending on the rigidity and elasticity of the contour) and an external rule (depending on the gradient of the image) until the contour can capture the glottal edges in the image. This deformation is performed through an energy optimization process, which aims to minimize the sum of the internal and external energy functions, corresponding to the contour shape and the image gradient, respectively [50]. The bottom and top lines detected from the unsupervised clustering method are provided to the developed ACM technique as the initial locations of the contours for the right and left vocal folds. The hybrid method was applied to all the kymograms at different intersections of the vocal folds for glottal edge detection. The detected edges in the kymograms were then registered back to the HSV frames for each vocalization.

## 3. Results

**Figure 1.**
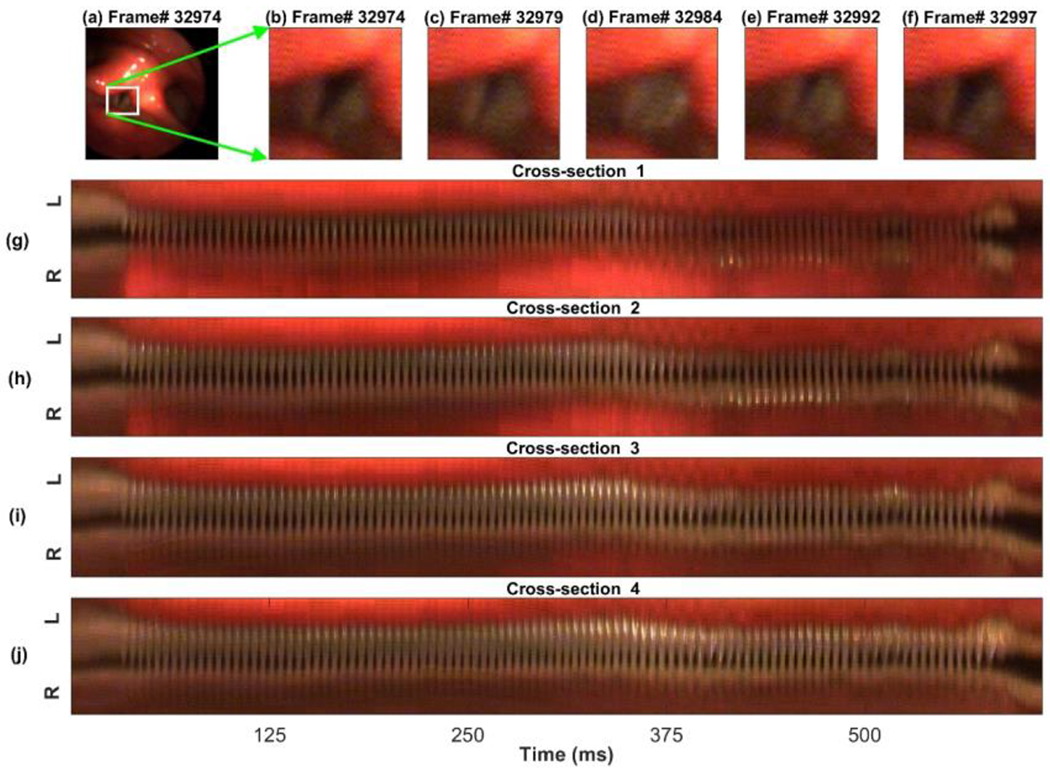
Panels (**b**–**f**): five HSV cropped frames for Frame 32,974, 32,979, 32,984, 32,992, and 32,997 after applying the motion window to five different HSV frames (one HSV frame is shown in Panel (**a**)). Panels (**g**–**j**): four extracted kymograms at different cross-sections of the vocal folds. The R and L on the y-axis indicate the right and left vocal folds in the HSV frames, respectively.

The following results demonstrate the implementation of the proposed method for the HSV data recorded from a vocally normal individual while reading the “Rainbow passage”. An example of five cropped HSV frames extracted from a vocalization after applying the temporal segmentation and motion compensation techniques is illustrated at the top of Figure 1. This vocalization was extracted between frames 32,709 and 35,061. The frame numbers are shown at the top of Panel (b)-(f). As seen, the motion window captures the size and the spatial location of the vocal folds during different phases of the vibratory cycle. After applying the motion window, the HSV kymograms were extracted at various cross sections of the vocal folds during each vocalized segment. Four kymograms, extracted at four different cross sections of the vocal folds during the same vocalization, are shown in Figure 1g–j. The y-axis of the kymograms represents the left-right dimension of the HSV frame, while the x-axis refers to the time (number of frames). Each kymogram in the figure displays the voicing onset and offset along with the vibration of the vocal folds.

**Figure 2.**
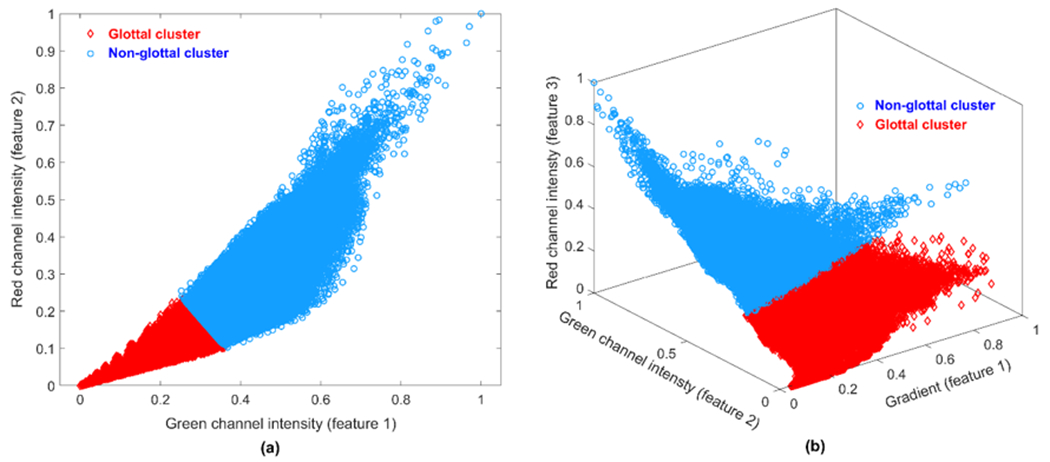
Scatter plots of the two clusters when applying the clustering method to the kymogram in Figure 1h between Frame 32,659 and 35,111: (**a**) using the green and red channel intensities as the features; and (**b**) using both green and red channel intensities along with the gradient as features.

**Figure 3.**
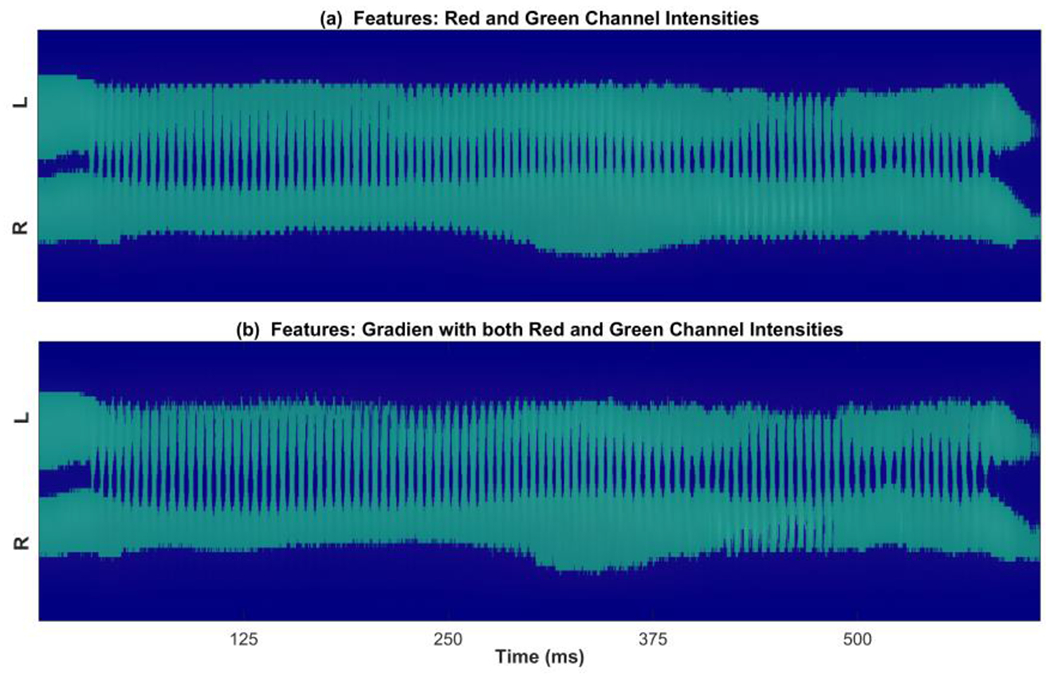
Clustered kymogram (from Figure 1h) by employing the k-means clustering algorithm using (**a**) the red and green channel intensities as features and (**b**) the gradient along with the red and green channel intensities as three features.

**Figure 4.**
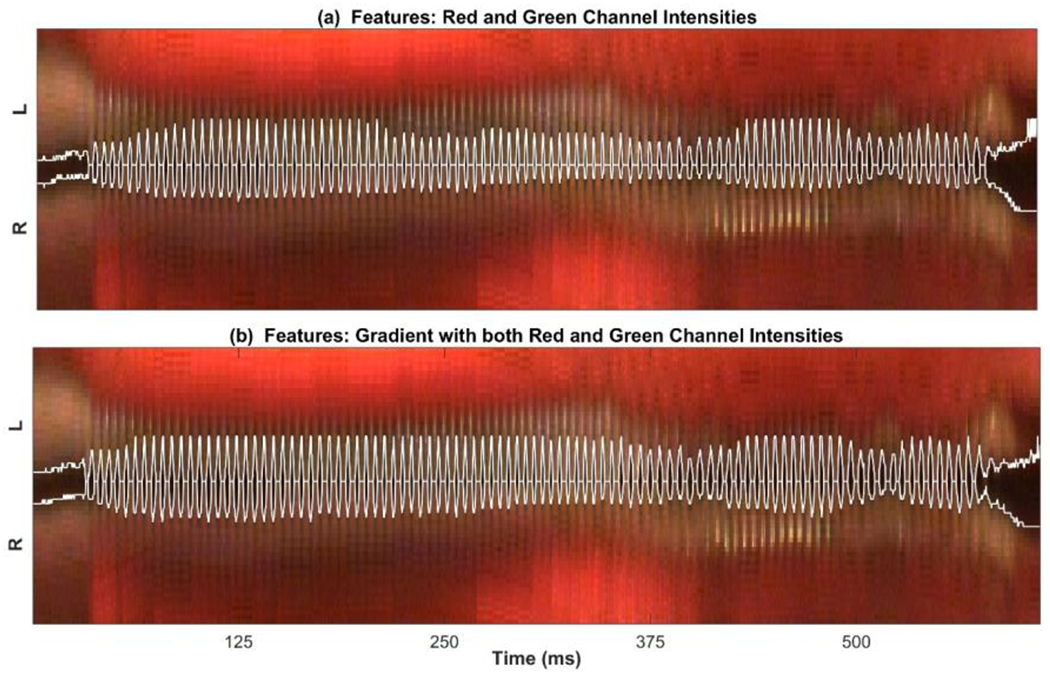
Detected glottal edges based on the results of the k-means clustering algorithm using (**a**) the green and red channel intensities as features and (**b**) using the gradient along with both red and green channel intensities as three features.

The k-means clustering technique was implemented for each kymogram. Different subsets of features were fed into the ML algorithm to determine the proper number and combination of features leading to an accurate vocal fold edge representation. Figures 2–4 illustrate a comparison between the results of applying two different combinations of the features for glottal area/edge detection: (i) red and green channel intensities as two features (Panel (a) in the figures) versus (ii) the image gradient along with the red and green channel intensities as three features (Panel (b) in the figures). The results of utilizing the other subsets of the aforementioned features to perform the clustering showed poorer performance of the method in comparison with using the selected feature combinations in Figures 2–4. Figure 2 shows the result of applying the clustering technique to the kymogram shown in Figure 1h between Frame 32,709 and 35,061 (for a total of 143,167 data points). The scatter plot in Figure 2a is generated by feeding the clustering algorithm the two intensity features: the green channel intensity and red channel intensity. The scatter plot in Figure 2b is generated using the gradient feature along with both red and green channel intensity features. The glottal area cluster in the kymogram is shown by red diamonds and the non-glottal cluster is shown by blue circles. As seen, after adding the gradient feature to the intensity features in Figure 2b, the two clusters can be distinguished in the scatter plot; in contrast, depending only on the intensity as a feature, it is relatively hard to divide the data points into two different clusters. The better performance of the ML method using the three features is more prevalent in Figure 3.

Figure 3 shows the two clusters after applying the k-means clustering technique to the kymogram in Figure 1h. The top figure illustrates the clustered regions using the two intensities as features and the bottom figure shows the result when using both the gradient and the intensities (green and red channel intensities) as three features. Figure 3 illustrates the clustered areas on the binary labeled kymogram so that only two distinct colors are shown representing the two clusters obtained. As seen, using the gradient in addition to the intensity features allows us to capture more information about the glottal area, which well aligns with the results obtained from the Figure 2.

Figure 4 shows the detected edges of the glottal area based on the results of clustering. In this figure, only the glottal cluster region is shown with a white line in the original kymogram (to have a better visual representation of the performance of the clustering method) using the intensity features (Panel (a)) and the gradient and intensity features (Panel (b)). The comparison of Panel (a) and (b) shows the improvement in clustering after adding the gradient feature to the intensity features. As can be seen in this figure, using only intensity features results in missing some spatial information about the glottal area, particularly during the sustained vibration of the vocal folds. On the other hand, the glottal edges were detected more accurately when the gradient feature was used along with both the red and green channel intensities. This improvement is more noticeable during the sustained oscillation of the vocal folds than during the voicing onsets and offsets.

**Figure 5.**
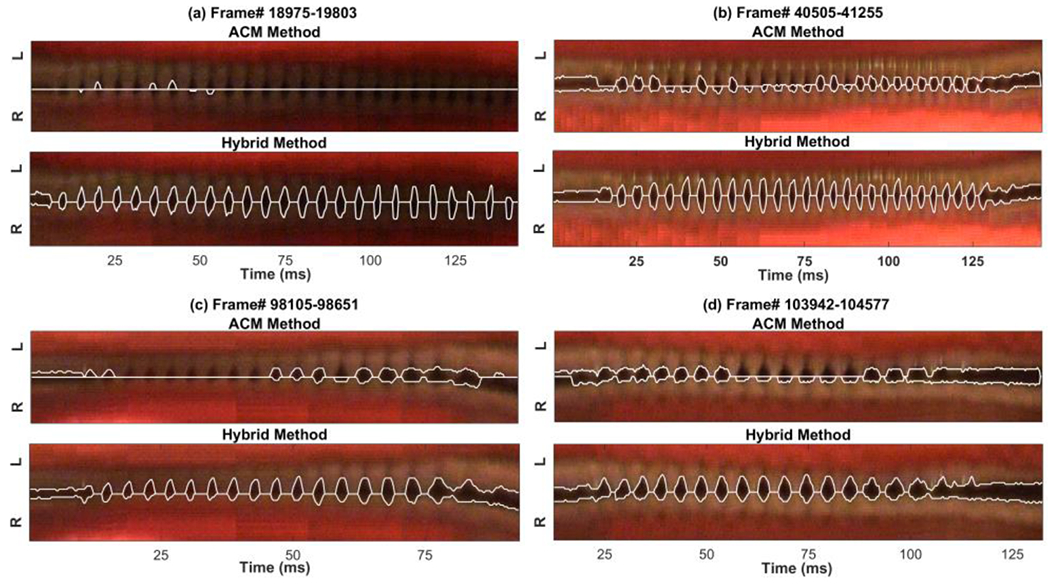
Detected glottal edges using the active contour modeling (ACM) method (top kymograms in Panel (**a**–**d**)) versus the hybrid method (bottom kymograms in Panel (**a**–**d**)) for the kymograms extracted at four different vocalizations.

The preliminary segmented glottal edges by applying the clustering technique were used as inputs to the ACM method. Figure 5 shows how using k-means clustering as an initialization step for the ACM impacts the accuracy of the method. The results are presented in four kymograms extracted at four different vocalizations. The detected glottal edges using the ACM alone and the developed machine-learning-based hybrid method are shown for two decent quality kymograms (between Frames 40,505 to 41,255 in Panel (b), and 103,942 to 104,577 in Panel (d)) and for two challenging kymograms (between Frames 18,975 to 19,803 in Panel (a), and 98,105 to 98,651 in Panel (c)). The figure depicts the result of applying the ACM method alone (the top figure in each panel) and the hybrid method at each vocalization (the bottom figure in each panel). Although the ACM performed better for the top kymograms in Panel (b) and (d) in comparison with the (more challenging) kymograms at the top of Panel (a) and (c), this method missed the glottal edges for several cycles as seen in the top figures in Panel (b) and (d). The ACM was not able to capture the glottal edges for many glottal cycles in the dim kymograms as seen in the top figures in Panel (a) and (c). In contrast, the hybrid method showed a considerable enhancement in the performance and high accuracy as it detected the glottal edges precisely for all the kymograms, as seen in the bottom kymograms in Panel (b) and (d), also in Panel (a) and (c) with an inferior quality and challenging kymograms.

**Figure 6.**
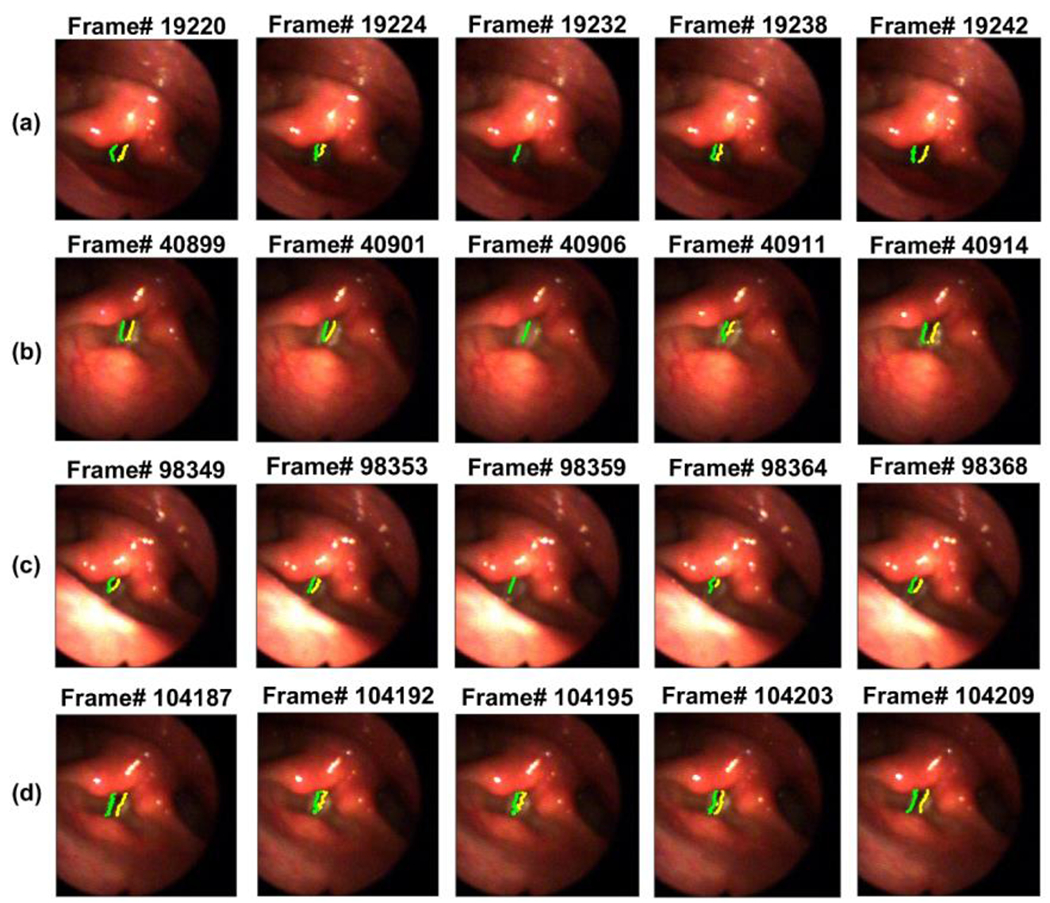
Five HSV frames from four different vocalizations (Panel (**a**–**d**): between Frame 18,975–19803, 40,505–41,255, 98,105–98,651, and 103,942–104,577) after applying the hybrid method and spatially registering the edges of the vibrating vocal folds.

In Figure 6, five HSV frames are presented from each of the four different vocalizations in Figure 5 along with the detected glottal edges by the hybrid method. This figure shows the captured edges after registering the glottal edges from the kymograms back to the HSV frames. For each vocalization, the five frames are chosen to show several frames from a different phase of a vibrating cycle of the vocal folds. As can be seen in Figure 6, the hybrid method was able to track the left and right vocal fold edges accurately during the vocal fold vibration in different frames and vocalizations.

## 4. Discussion

The temporal segmentation and motion compensation algorithms were successful in capturing the location of the vibrating vocal folds in a cropped motion window, which prepared the HSV frames for kymogram extraction. The HSV kymograms were generated at different cross sections of the vocal folds during each vocalization. The moment of inertia was used to successfully determine a horizontal line spanning through the center of the vocal folds in each kymogram, which was an important step before applying the hybrid spatial segmentation method to the kymograms.

The selection and extraction of the appropriate features were done in order to implement the unsupervised ML technique (i.e., k-means clustering). A different number and combination of features were fed into the ML algorithm to determine the salient subset of features for the development of the method. These features included the intensities of red and green channels and the image gradient. It was found that using these three features was the most appropriate combination of features in terms of obtaining an adequate clustering performance. Given the three considered features, the implemented clustering algorithm was able to precisely cluster the kymograms into two clusters (glottal area and non-glottal area). Subsequently, the edges of the clustered glottal area were spatially segmented, returning the top and bottom initialization contour lines corresponding to the left and right vocal folds, respectively.

After obtaining the initial contours from the clustering technique, they were used as inputs to the ACM method to enhance its performance in segmenting the vocal fold edges. The ACM method was successfully applied to the kymograms utilizing the initialized contours. The main weakness of the ACM method is the sensitivity to the contour initialization, which should be selected to be close to the glottal edges. In this study, using the clustering technique to initialize the active contours significantly improved the accuracy of the hybrid ACM in comparison with using the ACM alone, as shown in Figure 5. This hybrid method allowed for the accurate representation of the edges of the vibrating vocal folds in the kymograms at different intersections of the vocal folds. Figure 5 illustrated a comparison between the new machine-learning-based hybrid method against the ACM alone in order to show to what extent the new hybrid technique enhanced the performance of the vocal fold edge representation in comparison with using only the ACM approach. The performance of the hybrid method was compared with that of the ACM by applying the two methods on two decent quality kymograms and two kymograms with dim lighting and degraded qualities. The results of the comparison revealed a significant improvement in edge detection by the hybrid method over using the ACM alone. This enhancement was more noticeable in the lower quality kymograms. This indicated how the proposed hybrid method was less vulnerable to the noise in the image compared to the ACM, which completely failed to detect the edges in the presence of significant noise in the kymograms. In addition, the computational cost of the hybrid method was half of the ACM technique.

After applying the hybrid method, the segmented edges in the kymograms, which were extracted at different vocal fold cross sections, were registered back to the HSV spatial frames to detect the vocal fold edges in each individual HSV frame. The performance of the proposed hybrid method was tested through visual inspection of the detected vocal fold edges in the HSV kymograms of different vocalization segments of the “Rainbow Passage.” Out of 76 vocalizations, the visual inspection of the detected vocal fold edges in the extracted kymograms demonstrated that the developed hybrid technique successfully captured the glottal edges for 74 vocalizations with an error less than ±1 pixel. This yields a high accuracy of 97.4% in vocal fold edge representation using the hybrid method for HSV data during connected speech. The only other study performing the same task that we can compare our work with was our previously developed ACM method [20], which detected the glottal edges accurately in 88% of the vocalizations in the same HSV sample. There are no other known studies of automated vocal fold segmentation of HSV recordings during connected speech. The current study presented several of the vocalizations, where the ACM method failed. The higher accuracy and performance of the hybrid method, as were shown in this study, reveals its superiority over the ACM method. The extracted kymograms of the two vocalizations in which the hybrid method did not perform accurately had extremely dim lighting across most of the frames, which also made the visual detection of the glottal boundaries impossible, making it challenging to create an accurate reference manually.

The hybrid method in this study is the first ML-based approach developed for vocal fold segmentation during connected speech. The recently developed deep learning approaches for vocal fold segmentation were all employed for HSV analysis during sustained vocalization with higher image quality [32–35]. The developed hybrid method is fully automated, while the deep learning techniques required manual labelling of a part of the dataset in order to train the deep neural networks. Moreover, the deep learning methods are all spatial segmentation techniques; however, the hybrid method in this study is a spatiotemporal method that would potentially lead to a higher robustness in case of irregular vocal fold closure. The hybrid method in this study relies on the accurate performance of the developed motion compensation method; however, this is not an issue with the HSV analysis during sustained vocalization due to the little change in the vocal fold location across frames. Since there is no known gold-standard accurate method to fully capture the vocal fold edges from HSV data during connected speech, visual inspection was performed to serve as reference for validating the performance of the developed technique. It should be noted that this study showed the feasibility of the hybrid method for vocal fold edge representation (in HSV data) during connected speech in one participant with no history of voice disorder. This method needs to be tested on more vocally normal participants, also on participants with voice disorders in order to be generalized.

Although the promising performance of the hybrid method was shown during vocal fold oscillation, the algorithm did not perform accurately before and after the onset and offset of vocal fold vibration. This was due to the deviation of the motion window from the vocal fold location before and after the oscillation. However, this did not contradict the purpose of the present study, which was to track the edges of the vocal folds during vocalization. In future, the development of an algorithm to automatically detect the edges of the vocal folds when adducted and not vibrating would be valuable in studying laryngeal maneuvers during connected speech. In addition, the proposed approach showed a promising performance for HSV data with the most challenging images, obtained by a color HSV system. This facilitates the future implementation of the proposed method on less challenging monochromatic images since a monochrome camera provides a higher sensitivity and dynamic range with better pixel representation. This will potentially lead to a higher accuracy and faster performance of the hybrid method for monochromatic HSV data. This study aimed to show the feasibility of this approach for color HSV images, which is preferred over monochromatic images by many voice specialists since color images allow them to better evaluate the health of the tissues.

## 5. Conclusions

Developing an automated technique for an accurate segmentation of the vocal fold edges from HSV is a crucial prerequisite for the objective analysis of vocal function during connected speech. In the present paper, a new automated technique was introduced to analytically represent the vocal fold edges from HSV data during running speech. The temporal segmentation and the motion compensation approaches used in this work successfully extracted the timestamps of the vocalized segments and localized the vibrating vocal folds of the HSV recording of the “Rainbow Passage”. Combining an unsupervised ML technique (i.e., k-means clustering) with an ACM approach resulted in a powerful hybrid method for spatial segmentation, which revealed a promising performance in precisely capturing the edges of the vocal folds across frames. This hybrid method helped overcome the limitations of the ACM approach in terms of addressing the dependency of ACM performance to contour initialization, enhancing the edge representation accuracy, mitigating the sensitivity towards image noise, and providing a lower computational cost. The proposed method demonstrated an encouraging performance for challenging HSV data obtained using a color camera—paving the path toward implementing the hybrid method on less challenging images (monochromatic images) with a higher accuracy and performance. Since the hybrid algorithm was automated, fast, and accurate, it can serve as a promising tool to facilitate the automated analysis and measurement of vocal fold dynamics, especially valuable with the challenges present in the endoscopic analysis of connected speech.
